# Ketenes in the Induction of the Methanol‐to‐Olefins Process

**DOI:** 10.1002/anie.202207777

**Published:** 2022-08-24

**Authors:** Xiangkun Wu, Zihao Zhang, Zeyou Pan, Xiaoguo Zhou, Andras Bodi, Patrick Hemberger

**Affiliations:** ^1^ Paul Scherrer Institute 5232 Villigen Switzerland; ^2^ National Centre of Competence in Research (NCCR) Catalysis Paul Scherrer Institute 5232 Villigen Switzerland; ^3^ Department of Chemical Physics University of Science and Technology of China Hefei 230026 China

**Keywords:** Ketene Chemistry, Ketene Methylation, Methanol to Olefins, Reaction Mechanism, Zeolites

## Abstract

Ketene (CH_2_=C=O) has been postulated as a key intermediate for the first olefin production in the zeolite‐catalyzed chemistry of methanol‐to‐olefins (MTO) and syngas‐to‐olefins (STO) processes. The reaction mechanism remains elusive, because the short‐lived ethenone ketene and its derivatives are difficult to detect, which is further complicated by the low expected ketene concentration. We report on the experimental detection of methylketene (CH_3_−CH=C=O) formed by the methylation of ketene on HZSM‐5 via operando synchrotron photoelectron photoion coincidence (PEPICO) spectroscopy. Ketene is produced in situ from methyl acetate. The observation of methylketene as the ethylene precursor evidences a computationally predicted ketene‐to‐ethylene route proceeding via a methylketene intermediate followed by decarbonylation.

## Graphical abstract

Operando synchrotron photoelectron photoion coincidence (PEPICO) spectroscopy identifies ketene and methylketene intermediates in methyl acetate conversion over the HZSM‐5 zeolite. The surface acetyl to olefins mechanism proceeds via methylation and decarbonylation processes.

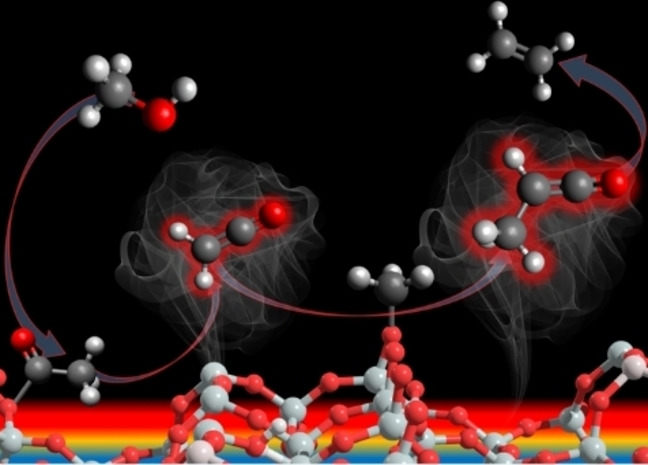

Methanol to olefin (MTO) conversion over zeolites has become a promising alternative to the oil‐based production of light olefins, i.e., ethylene and propylene.^[1]^ Predictive kinetic models for MTO processes require a detailed understanding of the underlying catalytic cycles to foster rational catalyst design and process optimization. It is generally accepted that MTO consists of two key steps: the development of the “hydrocarbon pool” by C−C bond formation from the C_1_ reactant is followed by an autocatalytic cycle.^[2]^ However, the exact mechanism of the first C−C bond formation in MTO remains elusive.^[3]^ Oxoniumylide, carbene, and methane‐formaldehydeylide type mechanisms were proposed in MTO, based on limited experimental evidence. However, the associated activation energies were calculated to be prohibitively high.[^3^, ^4^] An alternative, including the carbonylation of methoxy species yielding methyl acetate (or surface‐bound acetyl) as the intermediate, was proposed by Lercher et al.^[5]^ The computed low‐energy carbonylation barrier and the facile formation of the methoxy group upon dissociative adsorption of methanol on zeolites makes this mechanism more feasible, which has also been supported by further experimental observations.[^3^, ^5^, ^6^]

The carbonylation of methoxy species can generate methyl acetate (MA), surface acetyl (SA) as well as ketene (**K**, CH_2_=C=O), and the importance of the latter has recently been highlighted by Chowdhury and Gascon.^[7]^ For a long time, the role of **K** could only be verified indirectly by co‐feeding D_2_O and observing doubly deuterated acetic acid (CH_2_DCO_2_D) as the product of the CH_2_CO+D_2_O reaction. Just recently, Cesarini et al. identified **K** directly in the oxygenate‐driven autocatalytic cycle of MTO using operando photoelectron photoion coincidence spectroscopy.^[8b]^ In addition to its role in MTO, **K** was experimentally observed by vacuum ultraviolet photoionization mass spectrometry (VUV‐PIMS) in syngas (CO/H_2_) to olefin conversion (STO).^[9]^ **K** can be readily protonated on acidic zeolites (e.g., HZSM‐5) to SA (CH_3_CO−) and form MA by methoxy addition in MTO (Scheme 1). The reverse reaction MA‐to‐**K** is equally feasible.^[10a]^ Therefore, although **K** and MA (or SA) were proven to be primordial C_2_ intermediates, it is not yet clear which of the two species represents the first C_2_ intermediate with a C−C bond (Scheme 1) on the way to the first olefin. Theory suggests that SA decomposes to **K**, followed by methylation to yield methylketene (MK), which yields ethylene after decarbonylation (Scheme 1).^[10]^ Propylene may be produced by additional methylation of MK into dimethylketene (DK), followed by decarbonylation (Scheme 1).[^10b^, ^10c^] The initially formed ethylene and propylene will then proceed over the olefin‐based autocatalytic cycle by methylation and cracking processes.^[11]^ Thus, while there is a consistent and viable proposal for the MTO and STO induction mechanism, its experimental validation remains a challenge because of the thermodynamic and kinetic instability of the key ketene intermediates.^[12]^

**Scheme 1:**
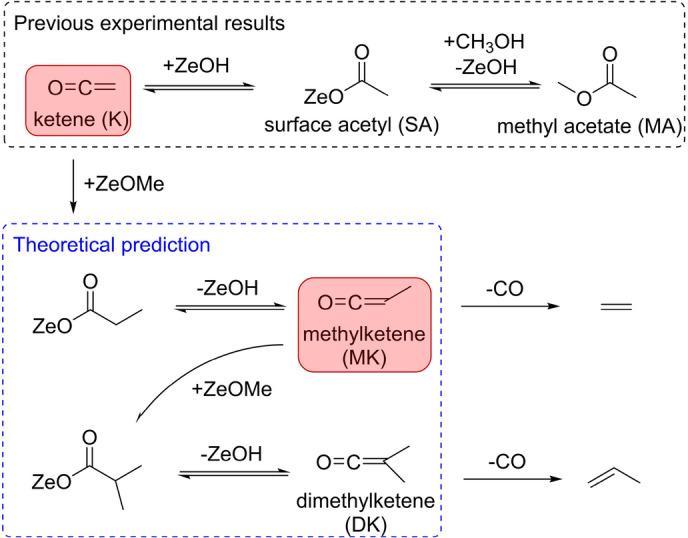
Experimental insights and theoretical predictions for the first olefin production on acidic zeolites derived from SA. Present findings are highlighted in red.

This conundrum motivated us to study MA conversion on HZSM‐5 and analyze the products in real time by operando synchrotron photoelectron photoion coincidence (PEPICO) spectroscopy to detect even trace amounts of short‐lived reactive intermediates.^[13]^ The selection of MA as the reactant is motivated by (1) the observation of MA as an intermediate in MTO[^3^, ^5^, ^14^] and (2) the proposed role of SA as a **K** source, which helps us achieve higher concentrations of **K**,^[15]^ thereby facilitating the detection of ketene‐mediated intermediates.

The PEPICO endstation^[16]^ and the reactor setup are described in the Supporting Information. In brief, the reaction is carried out in a heated quartz microreactor loaded with HZSM‐5. The continuous gas flow from the reactor, containing products and reactive intermediates, expands into vacuum to form a molecular beam, which freezes out the chemistry and suppresses quenching. The sample is ionized using vacuum ultraviolet synchrotron radiation. Photoelectrons and ‐ions are detected in coincidence, which permits us to measure mass spectra as well as photoion mass‐selected threshold photoelectron spectra (ms‐TPES) for the isomer‐specific assignment of each *m/z* peak. Blank experiments (Figure S1) without catalyst do not show reaction products below 450 °C. Figure 1 shows photoionization mass spectra of MA conversion over commercial HZSM‐5 as a function of the reaction temperature. At 180 °C, only MA is seen at *m/z* 74. At 250 °C, peaks grow in at *m/z* 46 and 58, identified as dimethyl ether (DME) and acetone, respectively. The ms‐TPES (Figure 2a) of *m/z* 42 at 250 °C can be unambiguously assigned to **K** via comparison with the reference spectrum, while propene and other olefins (C_*n*_H_2*n*_) (Figure 1) were not observed. This suggests that **K**, DME, and acetone are early intermediates in the MA‐to‐olefins process. Acetone is also an intermediate derived from SA (Scheme 2),^[17]^ which can convert to **K** over the zeolite, and an equilibrium is likely to be formed promptly in the MTO process.^[18]^ However, it is more likely to be converted to olefins by the decomposition of its aldol condensation products.^[19]^ DME‐to‐olefins share the reaction mechanism with MTO, as DME is an observed MTO intermediate obtained via the dehydration of methanol.[^5^, ^20^] The carbonylation of methoxy groups derived from methanol and DME to form MA and its derivatives was reported as the first C−C bond generation process in MTO.^[5]^ Overall, **K** can be produced from MA on HZSM‐5 at a relatively low temperature of 250 °C prior to olefin production.

**Figure 1:**
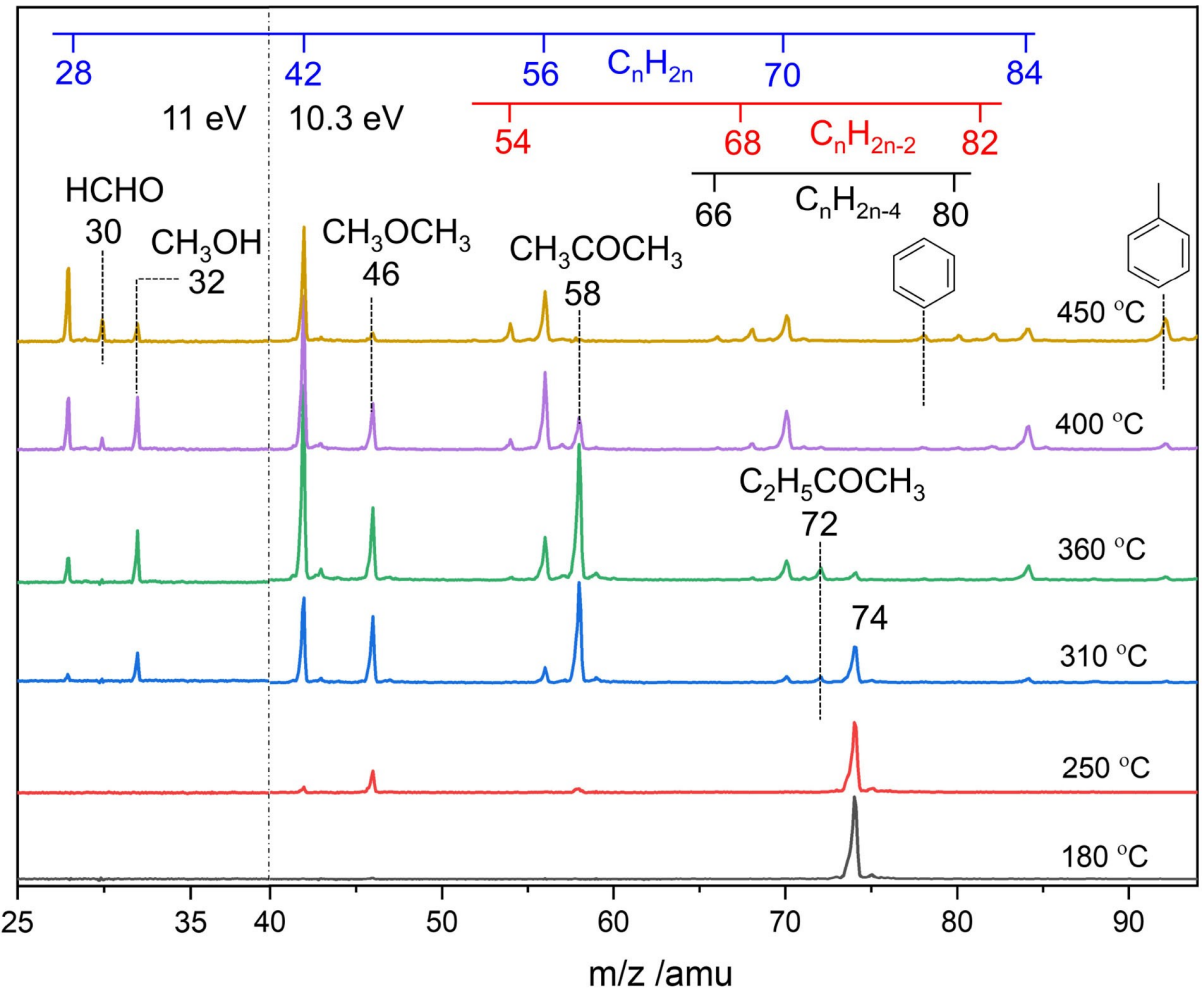
Photoionization mass spectra of MA conversion over HZSM‐5 at 10.3 eV photon energy (*m/z*>40) and 11 eV (*m/z*<40). Reaction conditions: 1 sccm 1 % MA in Ar, 19 sccm Ar, ≈400 mbar pressure, 10 mg HZSM‐5 with 1 cm catalyst length.

**Figure 2:**
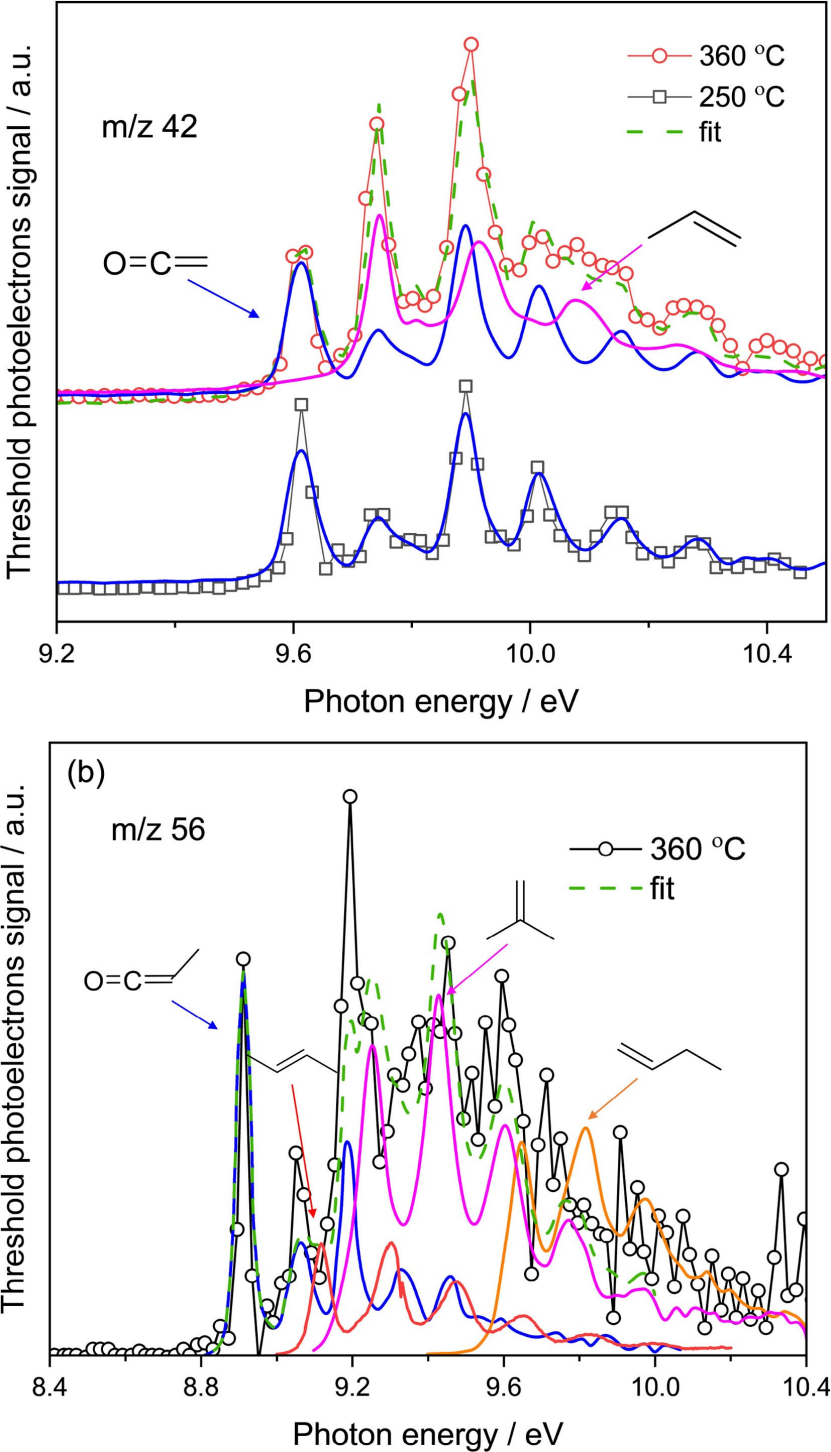
ms‐TPES of a) *m*/*z* 42 and b) *m*/*z* 56 in MA conversion over HZSM‐5. Same reaction conditions as in Figure 1. **K**, MK, and butene reference spectra are obtained from the literature,^[23]^ propene was measured herein.

**Scheme 2:**
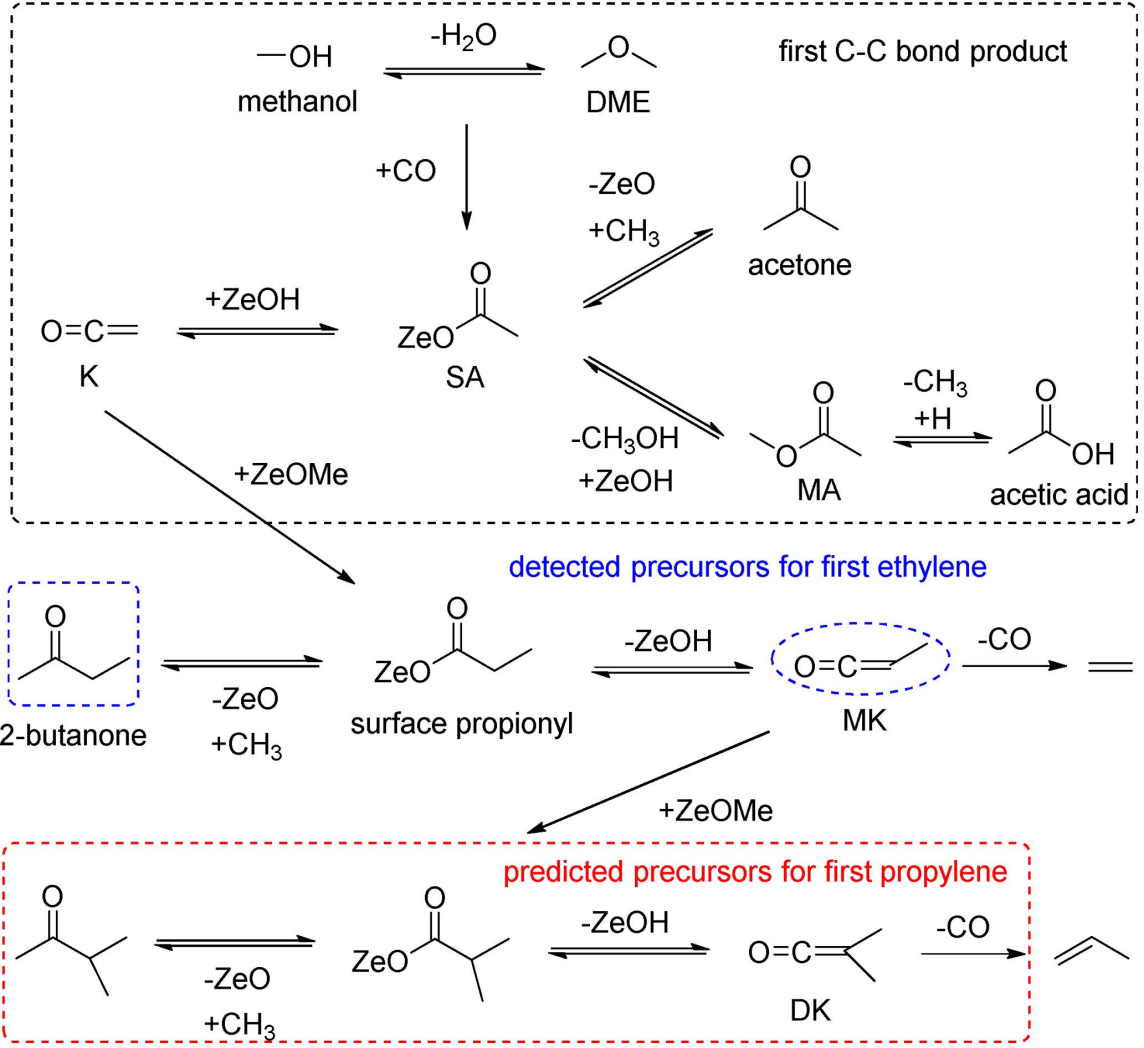
Proposed formation routes for (top) the first product with a C−C bond, (middle) first ethylene, and (bottom) first propylene based on SA on acidic zeolites. Molecules in blue ellipses are the detected precursors for ethylene. Molecules in the red rectangle are predicted precursors for propylene.

To understand how **K** transforms into olefins on HZSM‐5, the reaction temperature was further increased. When heating to 360 °C, the DME and acetone peaks increase at the expense of the MA peak (Figure 1), indicating that more MA is converted into DME and acetone. At the same time, singly unsaturated olefins (C_*n*_H_2*n*_) are also observed, including ethylene (*m/z* 28), propylene (*m/z* 42), butylene (*m/z* 56), pentene (*m/z* 70), and hexene (*m/z* 84). This is characteristic of the onset of the olefin‐based cycle.^[21]^ By further increasing the reaction temperature to 450 °C, the DME and acetone signals drop as further olefins and aromatics are formed, e.g., butadiene (C_4_H_6_, *m/z* 54), cyclopentadiene (C_5_H_6_, *m/z* 66), pentadiene (C_5_H_8_, *m/z* 68), benzene (C_6_H_6_, *m/z* 78), cyclohexadiene or methycyclopentadienes (C_6_H_8_, *m/z* 80), hexadiene (C_6_H_10_, *m/z* 82), and toluene (C_7_H_8_, *m/z* 92). Xylene (C_8_H_10_, *m/z* 106) and heavier olefins were also observed at high temperatures at *m/z* 94, 96 etc., as shown in Figure S2. In addition, a weak signal is found at *m/z* 32 at 250 °C and becomes more pronounced at higher temperatures (Figure 1), identified unambiguously as methanol based on the ms‐TPES at 250 °C (Figure S3). The simultaneous appearance of methanol and **K** at 250 °C is indicative of a MA‐to‐**K** process through SA (Scheme 2), because methanol is a co‐product in the MA‐to‐SA reaction. Since SA is also observed as the first C_2_ intermediate in MTO,^[5]^ our MA‐to‐olefins study mimics the MTO chemistry well. Formaldehyde (HCHO, *m/z* 30) and methane (*m/z* 16) are only observed above 400 °C, as shown in Figure 1 and Figure S4. It is commonly accepted that formaldehyde is formed via the disproportionation of methanol on acid sites^[4b]^ and may react with olefins in a stepwise manner to form aromatics on the acid sites of the zeolite.^[22]^ This is rationalized in our experiments by the appearance of both HCHO and aromatics in sync above 400 °C (in Figure 1 and Figure S4). As the HCHO formation temperature is higher than the ketene formation and olefin‐cycle temperatures, the methane‐formaldehyde route is suppressed at our conditions, enabling us to selectively probe the ketene associated routes. We now focus on the relatively low reaction temperature of 360 °C to further identify a second elusive ketene derivative by ms‐TPES and elucidate its role in the formation of singly unsaturated olefins, such as ethylene.

In contrast to **K** being the sole carrier of the *m/z* 42 ms‐TPES at 250 °C (Figure 2a), both **K** and propylene are detected at 360 °C, which exemplifies the ketene to olefin route in the induction phase of the olefin‐based catalytic cycle. When analyzing the *m/z* 56 ms‐TPES recorded at 360 °C (Figure 2b), we found strong evidence for MK, due to its sharp and characteristic origin transition at its ionization energy of 8.95 eV, the lowest among the C_3_H_4_O isomers and in agreement with the literature reference spectrum.^[23b]^ Above 9 eV, the MK ms‐TPES is overlapping with that of the isobaric C_4_H_8_ isomers 1‐butylene, 2‐butylene, and iso‐butylene.^[23c]^ To the best of our knowledge, this is the first time MK is observed in the context of the MTO reaction network. Additionally, the *m/z* 72 signal found at 360 °C can be assigned to 2‐butanone (C_2_H_5_C(=O)CH_3_, Figure S5). 2‐butanone is derived from surface propionyl (C_2_H_5_CO−) similarly to the SA to acetone route (Scheme 2). Analogously, surface propionyl may then desorb from the surface to form MK (Scheme 2), thus, the 2‐butanone signal represents a further piece of indirect evidence for the MK/surface propionyl/2‐butanone equilibrium. When the temperature is further increased to 380 °C, the MK signal decreases (Figure S6), as the ethylene (*m/z* 28, Figure S7) increases, suggesting that MK likely decarbonylates to ethylene. Thus, we propose that our unambiguous identification of MK verifies the previous theoretical prediction that ethylene is produced by methylation of **K** and subsequent decarbonylation in the induction period of MTO over zeolites (Scheme 2).[^10a^, ^10b^] Analogously, based on these calculations, dimethylketene DK was proposed as the precursor for propylene. However, the *m/z* 70 ms‐TPES can be fully assigned as pentene (C_5_H_10_) and DK was absent (Figure S8) at its ionization energy of 8.37 eV (G4). This may be rationalized by its high reactivity: The 0 K decarbonylation energy of MK to yield ethylene is 1.7 kJ mol^−1^ according to enthalpy of formation data from the Active Thermochemical Tables.^[24]^ This can be compared with our G4‐calculated decarbonylation energies of MK and DK of −3.4 and −9.5 kJ mol^−1^ (**Table S1**), respectively. Furthermore, the calculated C=C bond energy in **K**, MK, and DK decreases from 353.4 through 307.8 to 273.6 kJ mol^−1^, which also correlates with the progressively increasing C=C bond length upon methylation. In addition, the C−CH_3_ bond energies are significantly higher (Table S1), which suggests that decarbonylation of MK and DK (Scheme 2) is preferred to demethylation in both. The weakest C=C bond in DK and its more exothermic decarbonylation energy suggests that DK is more readily decarbonylated than MK. Furthermore, the 0 K reaction energy of the gas‐phase **K** methylation (60.5 kJ mol^−1^) is higher than that of MK (49.5 kJ mol^−1^) based on our calculations (Table S1). Note that H will likely be bound stronger on ZSM‐5, which lowers the effective methylation reaction energy, but the surface trend is expected to be consistent with the gas‐phase result. The high reactivity of DK may result in an even lower expected concentration on the catalyst surface than that of MK. Thus, DK likely desorbs as propylene after decarbonylation, which explains why the DK ms‐TPES stays below the detection limit in Figure S7.

Based on our experimental results and supported by computations, we have summarized the reaction mechanism in Scheme 2. The first C−C bond formation in early‐stage MTO proceeds via the SA and **K** intermediates.[^3^, ^5^, ^25^] SA can be formed by carbonylation of methoxy species derived from methanol and DME. Due to the relatively low carbonylation rate, the obtained concentration of SA and ketene‐derived intermediates, e.g., MA, acetic acid, acetone, is low, leading to low **K** concentrations. We selected MA as reactant and utilized operando PEPICO spectroscopy to boost sensitivity towards short‐lived reactive intermediates. By analyzing the temperature‐dependence of the mass spectra and assigning MK and 2‐butanone, we found compelling experimental evidence for the **K**‐to‐ethylene route on zeolites based on the presence of MK, which subsequently decarbonylates (Scheme 2). This proves the theoretical prediction of Plessow and Studt experimentally.[^10a^, ^10b^] The second methylation step to form DK, which then decarbonylates to propylene could only be verified indirectly, likely due to the low DK concentration and faster decarbonylation rate due to the weaker C=C double bond (Table S1). However, it is reasonable to assume that methylation of MK will occur because of the lower reaction energy than that of **K**.

In summary, methyl acetate was used as reactant to generate surface‐bound acetyl and ethenone ketene, i.e., the first C−C bond intermediates in the induction stage of the MTO process. Ketene derived from surface‐bound acetyl was experimentally detected at temperatures as low as 250 °C by operando PEPICO spectroscopy. Methylketene was detected as a secondary intermediate at higher reaction temperatures. This provides first experimental evidence for the computationally predicted ketene‐to‐ethylene route, which proceeds through methylation and subsequent decarbonylation on acidic zeolites. The detection of methylketene fills a long‐standing knowledge gap on how the initial C−C bond intermediates convert to light olefins. In addition to methylketene, 2‐butanone was also observed, which represents indirect evidence for surface‐bound propionyl species. Although dimethylketene was not observed experimentally, it is reasonable to assume that it is formed by further methylation of methylketene based on thermodynamic arguments. Nascent dimethylketene likely readily decarbonylates to propylene and hardly leaves the catalyst surface, which explains why it evaded detection.

## Dataset:

Data presented in the main figures of the manuscript are publicly available through the repository: https://doi.psi.ch/detail/10.16907%2F4cb6c13f‐9bd9‐48be‐8288‐728b038f2b58.

## Conflict of interest

The authors declare no conflict of interest.

## Supporting information

As a service to our authors and readers, this journal provides supporting information supplied by the authors. Such materials are peer reviewed and may be re‐organized for online delivery, but are not copy‐edited or typeset. Technical support issues arising from supporting information (other than missing files) should be addressed to the authors.

Supporting Information

## Data Availability

The data that support the findings of this study are openly available in PSI Public Data Repository at https://doi.psi.ch/detail/10.16907%2F4cb6c13f‐9bd9‐48be‐8288‐728b038f2b58.
