# Supplementary material for: Ketenes in the Induction of the Methanol‐to‐Olefins Process
Source: Angew Chem Int Ed Engl. 2022 Aug 24;61(41):e202207777. doi: 10.1002/anie.202207777 (PMC9804150; doi:10.1002/anie.202207777)
Supplement: Supplementary file 1 — Supporting Information [file ANIE-61-0-s001.pdf]

Angewandte Chemie
Eine Zeitschrift der Gesellschaft Deutscher Chemiker

Supporting Information

Ketenes in the Induction of the Methanol-to-Olefins Process

*X. Wu, Z. Zhang, Z. Pan, X. Zhou, A. Bodi, P. Hemberger**

Experimental and computational methods

Methyl acetate (MA, Sigma–Aldrich, 99%) conversion experiments were carried out on the double imaging photoelectron photoion coincidence (CRF-PEPICO) spectrometer at the VUV beamline of the Swiss Light Source, Paul Scherrer Institute.[1-2] Dispersed bending magnet synchrotron radiation was focused at a 200 µm exit slit in a differentially pumped rare gas filter with a mixture of noble gases (Ne:Ar:Kr 6:3:1) at a pressure of 10 mbar over an optical length of 10 cm to suppress higher-order radiation above 14 eV. The photon beam entered the experimental chamber at the ionization region ca. 50 cm downstream after the focus. The absolute photon energy was calibrated using autoionization lines of argon in the first and second order.

MA was diluted with Ar into a 2 L stainless steel bottle at a concentration of 1%. Then, 1 sccm 1% MA/Ar together with 9 sccm Ar was fed into the quartz reactor (4 mm outer diameter, 2 mm inner diameter, 26 mm heated length, 1 mm nozzle). The reactor was packed with ~10 mg HZSM-5 catalyst, and glass wool was used to immobilize the catalyst. HZSM-5 is obtained by calcination of commercial ammonium ZSM-5 (Si/Al = 25).[3] A cylindrical wire heater (4 mm inner diameter) connected to a DC power supply (Voltcraft) was applied to heat the reactor, and the reactor temperature was monitored by a type K thermocouple attached to the outside reactor wall in the middle of the reactor. The inside temperature of the reactor was calibrated against the outside temperature, and temperatures shown in the manuscript refer to the actual temperature of catalyst bed. Reactants and products desorbed from the catalyst surface left the reactor and formed a molecular beam in high vacuum, which was then skimmed using a 2 mm diameter skimmer placed before the ionization chamber. Reactive collisions were suppressed in the expansion and reactive intermediates are preserved. VUV radiation ionizes the gas mixture, the produced electrons and ions were extracted in opposite direction by a 216 V cm^{-1} constant electric field, and both were detected in velocity map imaging conditions by position-sensitive delay-line anode detectors (Roentdek, DLD40). As the electron time of flight (TOF) is negligible relative to the ion TOF, electron hits can be used as the start signal for the ion TOF analysis.[4] Threshold electrons with less than 5 meV kinetic energy and kinetic electrons without an off-axis momentum component are projected together onto the central spot of the detector. To obtain the threshold ionization signal, the hot electron contamination of the center signal was subtracted based on the signal in a small ring around the

center spot as proposed by Sztáray and Baer.[5] ms-TPE spectra were acquired by scanning the photon energy in steps of 20 meV, which are used for the isomer selective detection of reactive intermediates and products, such as ketene and propylene.

Quantum chemical calculations were performed using Gaussian 16.[6] Optimized geometries for reactants, intermediates and products were obtained using density functional theory at the B3LYP/6-311++G(d,p) level. Franck–Condon simulations were carried out in the double harmonic approximation including the Duschinsky rotation. The relevant products were re-calculated using the G4 composite method to obtain more accurate energetics.[7]

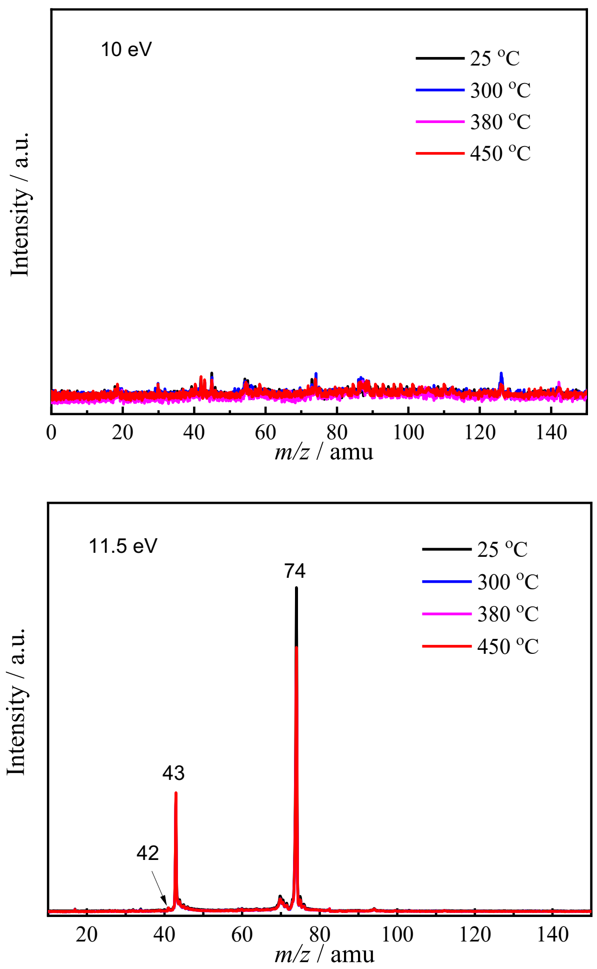

Figure S1. Photoionization mass spectrum from MA conversion in blank experiment at 10 eV and 11.5 eV.

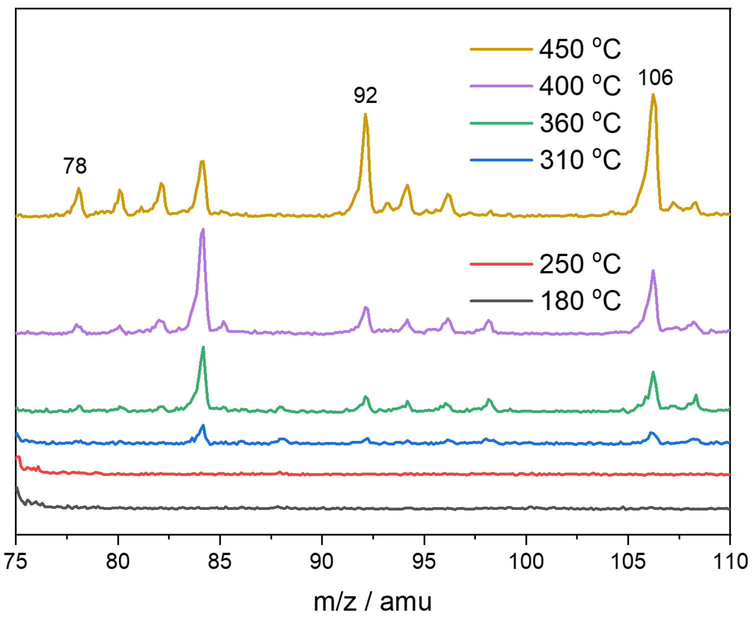

Figure S2. Photoionization mass spectrum from MA conversion on HZSM-5 recorded at a photon energy of 10.3 eV.

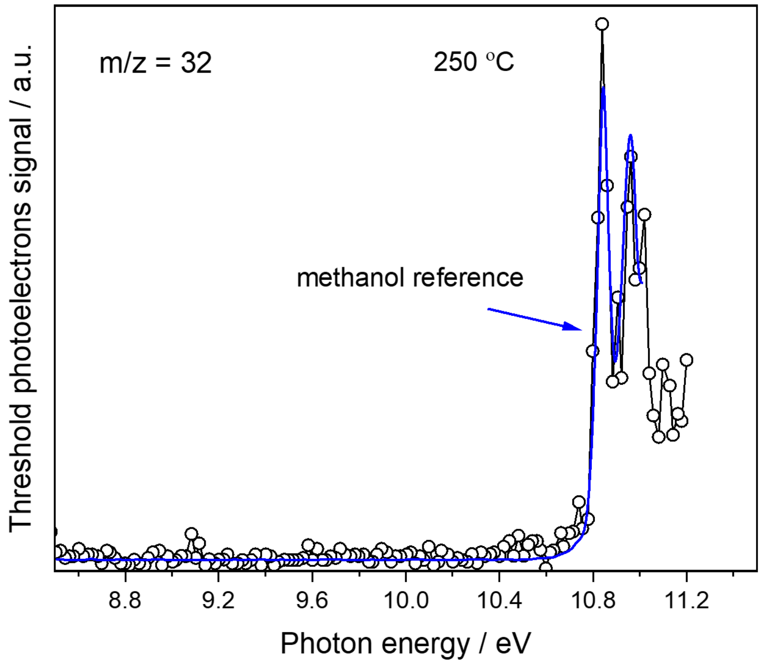

Figure S3. Photoion mass-selected threshold photoelectron spectrum (ms-TPES) of *m*/*z* 32 in MA conversion over HZSM-5 at 250 °C plotted together with a methanol reference *m*/*z* 32 ms-TPES. Methanol reference was measured herein.

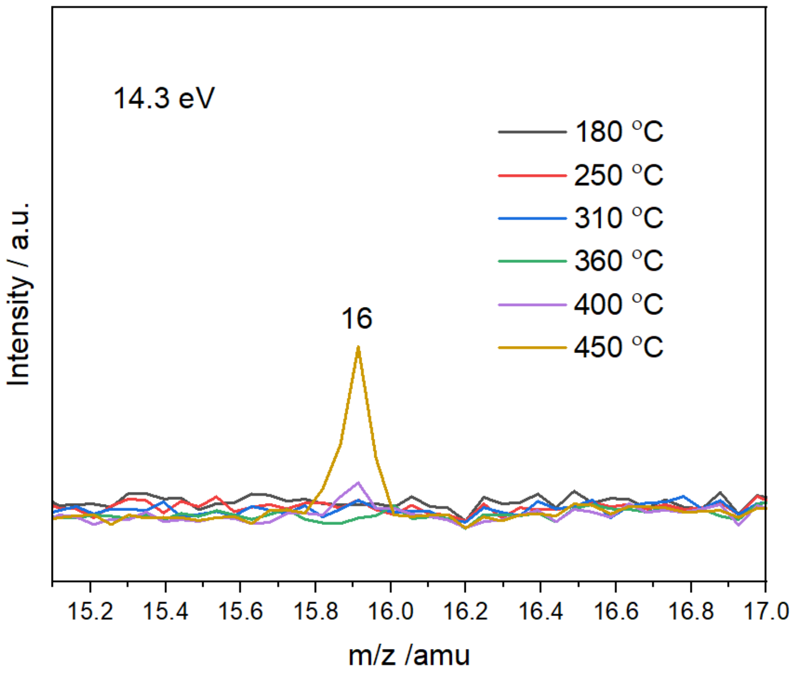

Figure S4. Photoionization mass spectrum of MA conversion on HZSM-5 recorded at a photon energy of 14.3 eV.

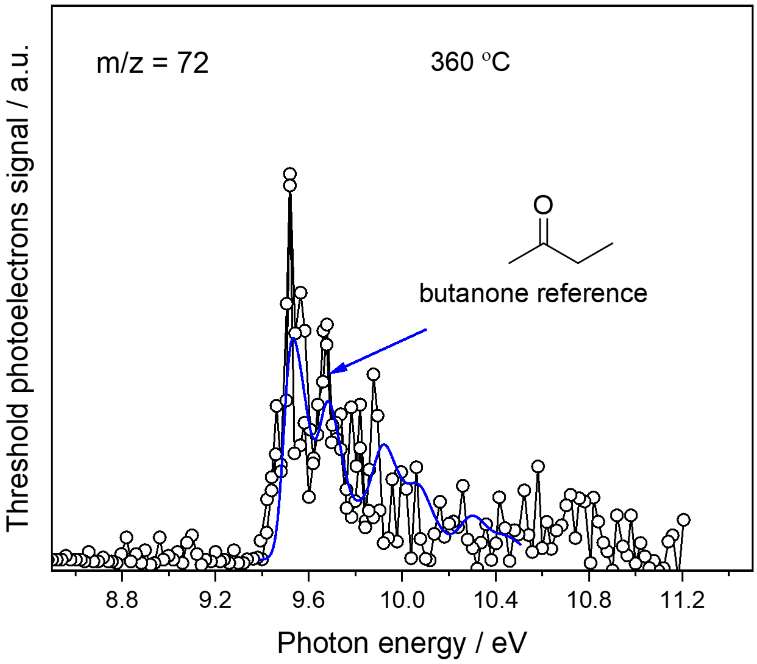

Figure S5. Photoion mass-selected threshold photoelectron spectrum of *m*/*z* 72 in MA conversion over HZSM-5 at 360 °C. The butanone reference spectrum is calculated based on a Frank–Condon simulation.

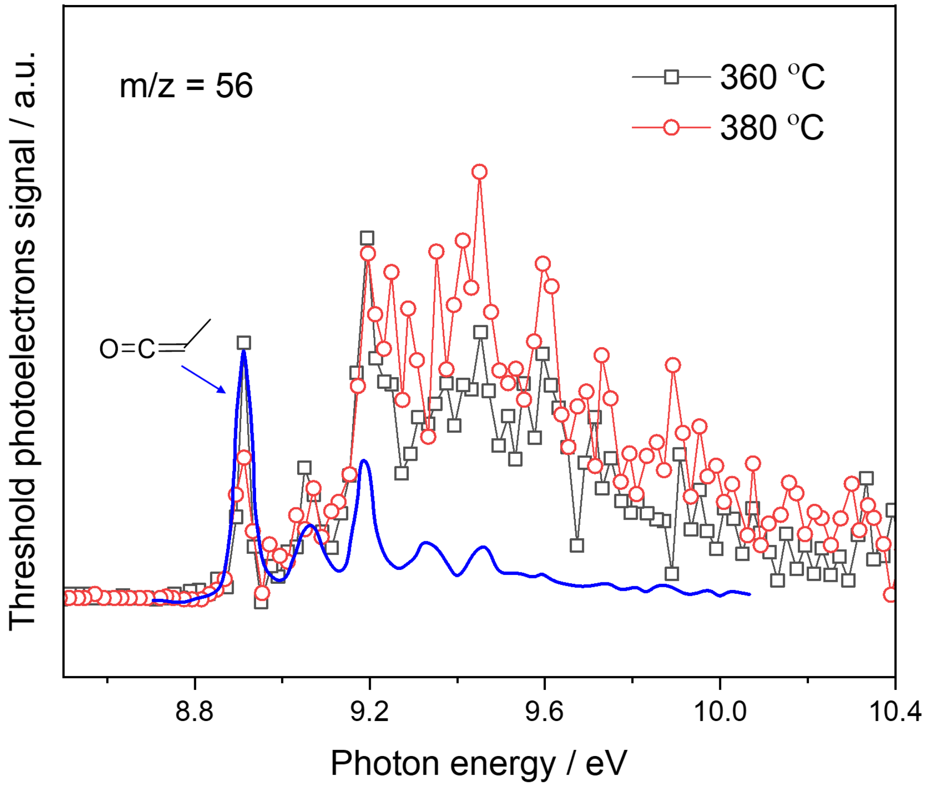

Figure S6. Photoion mass-selected threshold photoelectron spectrum of *m*/*z* 56 in MA conversion over HZSM-5 at 360 and 380 °C. The feature at 8.95 eV can be assigned to methylketene which clearly decreases at 380 °C in comparison to the butene isomers, which are ionized above 9.1 eV. The methylketene spectrum (blue line) is taken from the literature.[8]

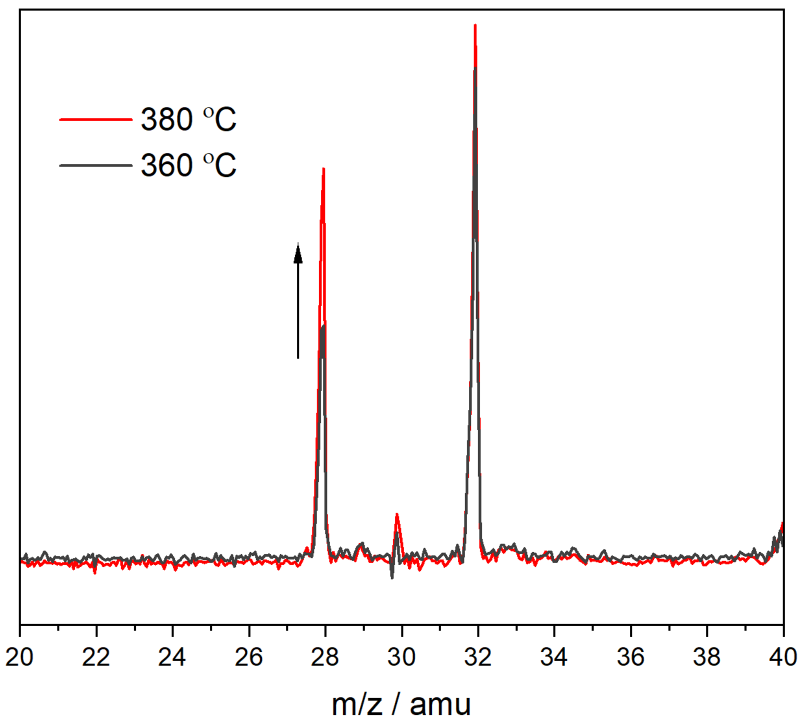

Figure S7. Comparison of photoionization mass spectrum from MA conversion on HZSM-5 at 360 and 380 °C recorded at a photon energy of 11 eV. The ethylene yield increases notably at 380 °C.

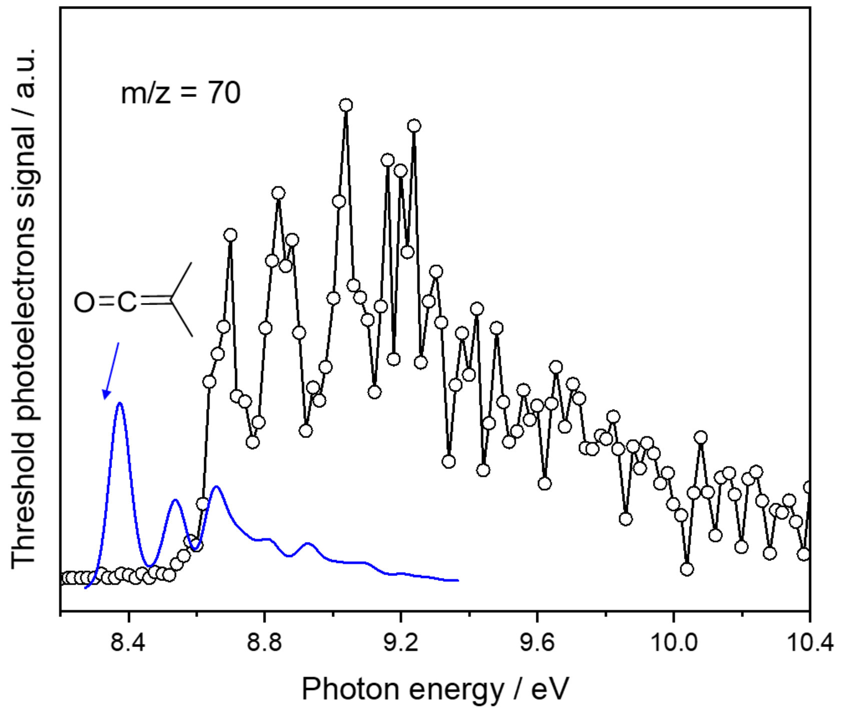

Figure S8. Photoion mass-selected threshold photoelectron spectrum of *m*/*z* 70 in MA conversion over HZSM-5 at 360 °C. The dimethylketene signal is below the detection limit based on the baseline signal at its ionization energy of 8.37 eV (calculated at G4 level of theory). The dimethylketene reference photoelectron spectrum is calculated based on a Frank– Condon simulation.

Table S1. Calculated homolytic C–C and C=C bond dissociation enthalpies at 0 K (kJ/mol), as well as the C=C bond length (Å) in ketene, methylketene, and dimethylketene; the decarbonylation energies (kJ/mol) of methyl- and dimethylketene to ethylene and propylene; 0 K methylation reaction energy (kJ/mol) of ketene and methylketene into methylketene and dimethylketene (R–H + Me → R–Me + H).

	$C–CH_3$	C=C (bond length)	decarbonylation $\Delta_r H_{0K}$	methylation $\Delta_r H_{0K}$
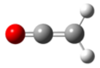		353.4 (1.308)		60.5
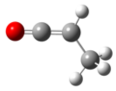	375.2	307.8 (1.309)	−3.4	49.5
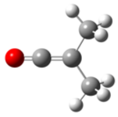	362.2	273.6 (1.311)	−9.5	

1. Johnson, M.; Bodi, A.; Schulz, L.; Gerber, T., Vacuum ultraviolet beamline at the Swiss Light Source for chemical dynamics studies. Nucl. Instrum. *Methods Phys. Res., Sect. A* **2009**, 610 (2), 597-603.
2. Sztáray, B.; Voronova, K.; Torma, K. G.; Covert, K. J.; Bodi, A.; Hemberger, P.; Gerber, T.; Osborn, D. L., CRF-PEPICO: Double velocity map imaging photoelectron photoion coincidence spectroscopy for reaction kinetics studies. *J. Chem. Phys*. **2017**, 147 (1), 013944.
3. Pan, Z.; Puente-Urbina, A.; Bodi, A.; van Bokhoven, J. A.; Hemberger, P. *Chem. Sci.* **2021**, 12, 3161-3169
4. Bodi, A.; Sztáray, B.; Baer, T.; Johnson, M.; Gerber, T., Data acquisition schemes for continuous two-particle time-of-flight coincidence experiments. *Rev. Sci. Instrum*. **2007**, 78 (8), 084102.
5. Sztáray, B.; Baer, T., Suppression of hot electrons in threshold photoelectron photoion coincidence spectroscopy using velocity focusing optics. *Rev. Sci. Instrum*. **2003**, 74 (8), 3763-3768.
6. Frisch, M. J.; Trucks, G. W.; Schlegel, H. B.; Scuseria, G. E.; Robb, M. A.; Cheeseman, J. R.; Scalmani, G.; Barone, V.; Mennucci, B.; Petersson et al., G. A., Gaussian 16 Revision A.03, Gaussian, Inc., Wallingford CT **2016**.
7. Curtiss, L. A.; Redfern, P. C.; Raghavachari, K., Gaussian-4 theory. *J. Chem. Phys*. **2007**, 126 (8), 084108.
8. Derbali, I.; Hrodmarsson, H.R.; Schwell, M.; Bénilan, Y.; Poisson, L.; Hochlaf, M.; Alikhani, M.E.; Guillemin, J.C.; Zins, E.L. *Phys. Chem. Chem. Phys*. **2020**, 22(36), 20394-20408.
